# An Ecological Approach to Smart Homes for Health Care Services: Conceptual Framework of a Smart Servicescape Wheel

**DOI:** 10.2196/12425

**Published:** 2019-02-08

**Authors:** Hyo-Jin Kang, Jieun Han, Gyu Hyun Kwon

**Affiliations:** 1 Department of Service Design Engineering Sungshin Women's University Seoul Republic of Korea; 2 Graduate School of Technology and Innovation Management Hanyang University Seoul Republic of Korea

**Keywords:** health care information management, system analysis, smart homes for health care services, ecological approach, conceptual framework, smart servicescape wheel

## Abstract

**Background:**

Smart homes are considered effective solutions for home health care for the elderly, as smart home technologies can reduce care costs and improve elderly residents’ independence. To develop a greater understanding of smart homes for health care services (SHHSs), this study accentuated the necessity of ecological approaches with an emphasis on environmental constraints. This study was based on 2 rationales: (1) users are inclined to perceive the service quality and service experience from environments (ie, servicescape) owing to the intangibility of health care and the pervasiveness of smart home technologies, and (2) both service domains are complex adaptive systems in which diversified and undefined service experiences—not only a few intended service flows—can be generated by complex combinations of servicescape elements.

**Objective:**

This study proposed the conceptual framework of a Smart Servicescape Wheel (SSW) as an ecological approach delineating the extensive spectrum of environmental constraints in SHHSs.

**Methods:**

The SSW framework was established based on a literature review.

**Results:**

Generally divided by perceptible and imperceptible servicescapes, the SSW consists of the perceptible Physical scape (ie, hardware components, environmental cues, and human states) and Social scape (ie, service relationships and social relationships) as well as the imperceptible Datascape (ie, computing intelligence, databases, and communication networks). Following the ecological approach, each category of the SSW is subdivided and defined at the level of components or functions.

**Conclusions:**

The SSW’s strengths lie in the various application opportunities for SHHSs. In terms of service planning and development, the SSW can be utilized to (1) establish the requirements for SHHS development, (2) associate with work domain analysis by defining component layers, and (3) understand the real contexts of SHHSs for the enhanced prediction of diverse service experiences. Regarding service management, it can be applied to develop measurement items for the operation and evaluation of SHHSs.

## Introduction

### Growing Needs for Informal Care for the Elderly

Increased life expectancy and declining birthrates have caused the rapid expansion of an aging population. Moreover, because of common chronic diseases (eg, heart disease, stroke, cancer, dementia, and diabetes), the demand and costs required for the care of elderly people are immensely growing [1,2]. To diminish these care costs in a relatively effective way, the solution focus is shifting away from formal care in hospitals and care centers toward informal care in personal home environments [3] because of the cost-effectiveness of informal care and less intrusiveness in one’s personal life [4,5]. Elderly people often require frequent and immediate medical interventions to prevent emergencies through continuous monitoring of their physiological parameters and activities [6]. This continuous monitoring, which might cause a significant financial burden in the case of formal inpatient care, can be realized more comfortably through informal care using a smart home platform [7]. Highly developed information and communication technologies can make home environments intelligent and provide remote, nonintrusive health care monitoring [6].

### Smart Homes as Effective Solutions for Home Health Care Services for the Elderly

Smart homes are being realized using ubiquitous computing and internet of things (IoT) technology, which connects devices and systems [8,9]. Previous studies have characterized the smart home as integrating technologies such as (1) home automation that allows devices and systems to be controlled automatically, (2) communication networks that connect the key electrical appliances and services, (3) remote access and control that allows the system to be operated at a distance, and (4) home intelligence that is aware of users’ individual contexts [10-13]. With those characteristics, scholars have defined the smart home’s purpose as providing a better home life experience by promoting safety, security, comfort, communication, and entertainment through technical management in the home environment [14,15]. Meanwhile, other scholars have pointed out the concerns over ethical and legal issues of smart home technologies regarding privacy, security, and confidentiality because of the highly identifiable nature of data [16-21]. Nevertheless, with a judicious approach to their ethical and legal risks, smart homes could be effective for continuous and remote monitoring of elderly health and for disease prevention, and they can reduce the costs of care for the aging generation while improving their independence and quality of life [2,3,22,23]. Various terms have been utilized to describe this technology, such as *health smart home* [24,25], *U-health smart home* [26], *ubiquitous health care* [6,27,28], and *smart homes in or for (elderly) health care* [2,3].

Meanwhile, the focus of previous research on smart homes has primarily been on the development and application of smart home technologies within the boundaries of computer science and engineering research [11,12,15,29-32] because smart home production is inherently technology-intensive [33]. Likewise, according to several literature reviews about health care in smart homes [2,3,22,34], smart homes for health care have largely been investigated from the perspective of technology application. Such studies have analyzed and classified literature on smart homes for health care according to the types of sensors, network or communication technologies, and the algorithm models of data processing [2,3,12].

However, health care services are cocreated by elderly residents’ experiences, and their experiences directly influence the perceived quality of the health care service [35,36]. Moreover, health care service experience is shaped by the interactions of elderly users with numerous touchpoints in the context of service [37]. In this sense, the viewpoints of service experience and service context are substantial in health care service, but they have not actively been engaged in research on smart homes for health care. Therefore, we accentuated the service experience and context perspectives of health care in smart homes in this study. To be consistent in our use of terminology, we adopted the term *smart homes for health care services* (SHHSs) and established an operationalized definition encompassing both technical and experiential viewpoints as follows: SHHSs are residences integrated with ubiquitous computing and IoT technology, which have the characteristics of home automation, communication network, home intelligence, and remote control and access by authorized health care personnel. They provide informal health care services such as real-time and long-term health monitoring, disease prevention by detecting anomalies, and unobtrusive activity support that does not interfere with individuals’ quotidian activities; thus, they reduce care costs, enable a satisfactory service experience in a private and comfortable home environment, and improve the independence of elderly residents.

### Why Do We Focus on Service Environments Regarding the Health Care Service Experiences in Smart Homes?

The academic perspective of service experience originates from service management research. In this field, the significance of service environments and service context engaged with service experience has been emphasized, as the service provision and service interactions occur in such environments and contexts [38]. First, *service interactions*, widely called *service encounters* [39] in service management research, are direct or indirect interactions between customers and service providers. Service interactions have been underscored because they can be diversified according to individual customers’ varied past experiences and preferences as well as probable differences between service providers [40]. Second, *service environment*, which Bitner defined as the *servicescape* [41], is the physical and social environment where the service interactions take place, and the servicescape can influence the different ways in which customers perceive and experience a service [41,42]. Finally, *service context* refers to the social and cultural structures, such as social norms and institutions, which could influence the way a service’s stakeholders interact with each other [43,44]. Therefore, service experiences are created by the service provisions and service interactions in association with diverse service environments (ie, servicescapes) and are concurrently profoundly influenced by service context [38]. In other words, service experience could be cocreated by multidimensional combinations of those 3 layers—service interactions, servicescapes, and service contexts.

The main focus of this study—the *servicescape* is a fundamental and componential level in the creation of service experiences as it can serve as an inducing or restricting factor on usage behaviors. Consequently, it can influence the service user’s emotional response, perception of the intangible service quality, satisfaction with the whole service experience, and intention of continuous usage [42,45-48].

In line with the significance of servicescapes in service experience, the servicescape perspective is also required for SHHSs, which has the characteristics of both health care services and smart services. Generally, health care is a credence-based service and patients tend to have difficulty assessing the technical quality of service (ie, the professional credibility of medical care) [36]. Instead, functional quality (ie, the service quality as it relates to the physical environment—the servicescape) is the primary determinant that affects patients’ perception of the health care service process [35,37]. Regarding smart services, intelligent devices and environments communicate and collect data in real time based on advanced networks and pervasive computing technology [49,50], but data transfer and technological elements are invisible or intangible to service users. Furthermore, direct interactions between users and service providers (ie, service encounters) are largely supplemented or replaced by interactions with smart devices and environmental elements. Consequently, users are inclined to perceive the service quality and service experience from the environments of smart services—namely, the *smart servicescape* [51]—thus, from the direct, visible, and tangible interactions with the smart servicescape. In this way, health care services and smart services commonly have a particular significance of servicescape from the viewpoint of service experience; for this reason, this study used the concept of smart servicescape for SHHSs.

The importance of the smart servicescape in SHHSs can be supported from another perspective; the ecological approach for a complex adaptive system. Rouse defined a *complex adaptive system* as a nonlinear and dynamic system that is composed of independent and intelligent agents whose behavior patterns emerge rather than being designed into or controlled by the system [52]. He also stated that one cannot force or command such systems to abide by behavioral and performance directions. The health care service falls under this system because of its diversified stakeholders and interests [53]. Meanwhile, the smart home can also be regarded as a complex adaptive system for 2 reasons. First, it is a dynamic and therefore complex system consisting of diversified intelligent agents (ie, smart devices and environments) based on real-time communication. Second, the smart home is an adaptive system that is continuously customized in accordance with the emerging behavioral patterns of its resident users because it is a private space where usage behaviors cannot be easily controlled to comply with service providers’ intended directions. The SHHS can, therefore, be considered a complex adaptive system.

### Ecological Approach to Smart Homes for Health Care Services

Then how can service experiences in a complex adaptive system such as an SHHS be understood? In the research field of service management or service design, service experiences have been analyzed and profiled, adopting *service blueprinting* or *customer journey maps*. Service blueprinting, pioneered by Shostack [54], is a diagrammatic approach in which the key activities and their linkages involved in service delivery are plotted. It clarifies the series of customer actions, physical evidence, and frontstage and backstage interactions to emphasize the perspective of service users [55,56]. Customer journey maps are also a diagrammatic method in which customers’ steps in engaging with multiple touchpoints in a service are illustrated [57]. These maps conceptualize service experiences as a chronological process of a customer’s journey with a service provider [58]. These approaches can provide an understanding of the sequential flow of tasks in a service itinerary (ie, the customer’s journey) that a service provider has intentionally designed. However, they may not be suitable for describing a complex adaptive system such as SHHSs, because diversified—hence unintended and undefined—behavioral patterns can be generated given the specificities of SHHSs (ie, multiple stakeholders, private home circumstances, and perpetual service duration for continuous health monitoring).

For this reason, the SHHS requires the *ecological approach*. This approach, which originated from Barker and Gibson’s ecological psychology theory [59,60], considers environmental and social factors as parts of a system. According to this approach, individuals are surrounded by a complex combination of physical and social variables that operate in both direct and indirect ways to influence human activities [61,62]. Vicente advocated for the ecological approach in the field of human-computer interaction (HCI) and claimed that a work analysis for complex sociotechnical systems needs to start with and prioritize environmental constraints [63]. Vicente classified the demands (or constraints) of human works into (1) *cognitive constraints* relevant to human cognitive systems such as mental models and (2) *environment constraints* that constitute the context in which humans are situated, such as their physical and social realities [63]. The dominant viewpoint in psychology and HCI has been the cognitivist approach, which emphasizes cognitive mental models such as the sequential flow of an instructional service itinerary. Alternatively, the ecological approach leads to the analysis of environmental constraints, and it enables individuals to understand the real context that may shape actual behaviors (ie, *behavior-shaping constraints*) [64] and allows them to deal with unexpected and variable situations [63]. Given that SHHSs are complex adaptive systems in which diversified and undefined behavioral patterns can be generated by complex combinations of contextual and environmental factors, the ecological approach is appropriate for understanding service experiences in SHHSs by identifying the smart servicescape. Considering the necessity of the ecological approach to SHHSs, this study proposed a conceptual framework of smart servicescape to delineate complex environmental elements of SHHSs.

### Fundamental Concepts for the Framework: Smart Servicescape

Ahead of investigating the smart servicescape, the concept of *servicescape* needs to be clarified. As briefly mentioned in the Introduction, Bitner [41] defined the servicescape as a man-made physical and social environment in which service encounters are framed. Bitner emphasized the effects of physical environments and classified them into 3 dimensions: (1) ambient conditions (circumstantial attributes, such as temperature, air quality, noise, music, and odor); (2) space and function (the arrangement and layout of the machinery, equipment, and furnishings); and (3) signs, symbols, and artifacts (visible communicators on the exterior and interior) [41]. Since then, many scholars have explored the categorization of servicescapes in diverse service sectors (eg, restaurants, leisure, hospitality, and health care facilities) [45,47,65-67]. Furthermore, the *social servicescape* has also been underlined including social or noncommercial relationships, such as direct interactions with service providers, indirect interactions with other customers, social density, and connectedness [42,68].

Founded on the notion of servicescape embracing physical and social aspects, we had proposed the concept of *smart servicescape* regarding smart services in a prior study [51]. Analyzing the cases of smart home service experiences, new dimensions, including *datascape*, were introduced to reflect the key characteristics of smart services (ie, real-time data collection and the continuous data exchange of intelligent objects) [51].

Meanwhile, several studies have been conducted on the architecture of smart homes in association with health care services [2,3,6,26]. Researchers have commonly proposed a four-layer architecture for SHHSs even though their detailed elements or labels differ. Generally, they have agreed on the layers of (1) sensors and actuators, (2) communication or network, (3) computing or data processing, and (4) services. The layer of sensors and actuators is relevant to physical devices, such as the units of home automation and home control, biosensors, and environment sensors. The communication layer refers to the wired or wireless home networking required for information gathering, service discovery, and appliance discovery. The computing and processing layer pertains to the information analysis and machine learning technology engaged in knowledge management and decision making. Finally, the service layer varies from the types of health care services to those of health care service providers and organizations. Although the layered architecture represents the overall structure of the SHHS well, the criteria for classification of the 4 layers are not clearly defined. Moreover, the aforementioned studies have been conducted in computer science and engineering fields; therefore, the research focus lies more on a technical perspective investigating which types of technologies are applied in each layer. The ecological approach has been absent from previous studies; particularly, the perspective of service experience and that of smart servicescape have not yet been investigated in SHHSs.

## The Smart Servicescape Wheel

### The Focus of the Smart Servicescape Wheel

**Figure 1:**
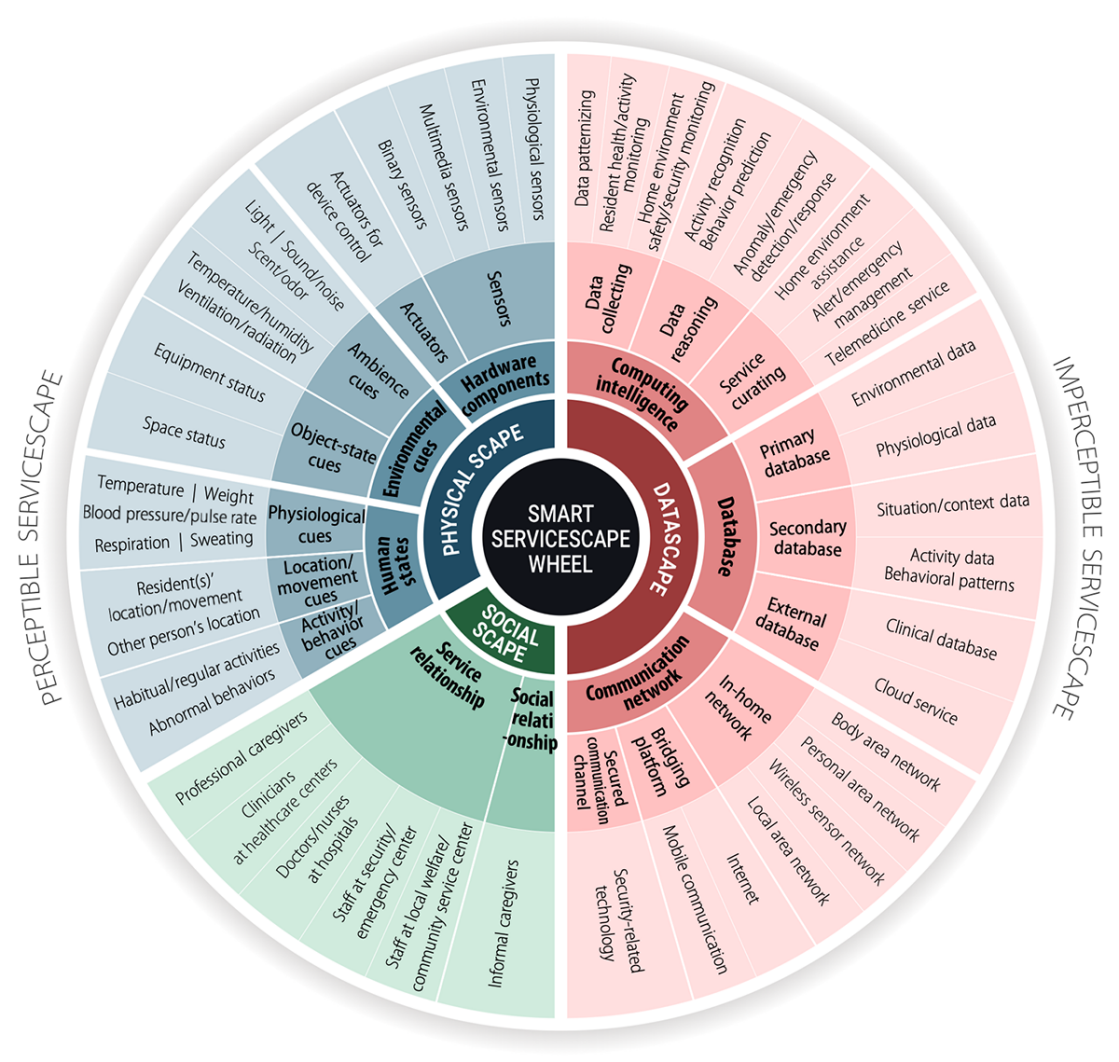
Smart servicescape wheel for smart homes for health care services.

To infuse the ecological approach with the understanding of service experiences in SHHSs—in other words, to delineate the extensive spectrum of environmental constraints in the complex adaptive system of SHHSs—we introduced a conceptual framework of the smart servicescape, the *Smart Servicescape Wheel* (SSW), as illustrated in Figure 1.

The Smart Servicescape Wheel for SHHSs globally classifies conventional servicescapes, such as the *Physical scape* and *Social scape* as *perceptible servicescapes*. The *Datascape*, which is a distinctive characteristic of smart services, is categorized as an *imperceptible servicescape* because its elements are hardly perceived by elderly residents unless they are intentionally visualized with certain service objectives. Moreover, the interactions that occur between the service user (ie, the elderly resident) and the perceptible servicescape can usually be recognized as *direct interactions*, such as the interactions of the elderly with smart devices (eg, physiological monitoring devices) or caregivers (eg, clinicians at health care centers and medical staff at hospitals). However, a large part of the interactions in the smart servicescape can hardly be perceived by the resident. These *ambient interactions* [69] are among the Datascape, Physical scape, and Social scape without the user’s direct intervention. Subsequently, the whole scope of direct and ambient interactions constitutes the *cyber-socio-physical interactions* [70].

Nevertheless, the focus of the SSW from the ecological perspective is not on the interactions diversely shaped in the spectrum of smart servicescape but on the detailed servicescape elements, namely the environmental constraints themselves. Moreover, the environmental constraints should be defined independently of any particular device, event, task, or interface because the ecological approach’s implication lies in designing a diverse spectrum of future service practices combining the environmental constraints rather than in designing a single flow of a service [63]. This implication implies that the smart servicescape elements need to be defined at the level of components or functions. Founded on this rationale, the Physical scape consists of (1) hardware components, (2) environmental cues, and (3) human states; the Social scape is composed of (1) service relationships and (2) social relationships; and the Datascape comprises (1) computing intelligence, (2) databases, and (3) communication networks. Accordingly, this section will discuss in detail each component of the SSW which is categorized in Table 1.

### Physical Scape

#### Hardware Components

The SHHS depends on data-collecting equipment and devices to monitor the state of residents and their environments [12]. At the level of components rather than devices, the hardware components consist of sensors and actuators [2,6,26]. Sensors are used to detect states and changes in the residents and their environments by measuring environmental or physiological parameters, and they are often seamlessly integrated into the living space and equipment [3,71]. Adopting the taxonomy of sensors by Amiribesheli et al [3], sensors are classified into (1) physiological sensors, (2) environmental sensors, (3) multimedia sensors, and (4) binary sensors.

First, physiological sensors are required to monitor a resident’s health condition. They acquire diverse biometric data (eg, body temperature, weight, blood pressure, pulse rate, blood glucose, and respiration—namely *physiological cues*, to be discussed in a later section) through various forms of medical equipment (eg, electrocardiography, electromyography, and electroencephalography) [3,12,72,73]. Second, environmental sensors are utilized to detect environmental data in smart homes, such as light, noise, temperature, and humidity—namely the *environmental cues* to be explicated in the following section. Light sensors that assess the illumination intensity and temperature sensors that measure data for heating or cooling air are commonly used [12,74]. Third, multimedia sensors include cameras and microphones to collect audiovisual data [3]. Audiovisual data enable the behavior monitoring and activity recognition of residents with high accuracy [75,76], but it involves privacy concerns [77]. Finally, binary sensors’ output data are in a discrete state of 0 or 1. These sensors are commonly used to detect the state of objects or residents because of the simple form of data and the unobtrusiveness in residents’ daily lives [3]. Common binary sensors include passive infrared sensors that collect data about residents’ movements or stillness [78], contact switch sensors that detect the state of objects (eg, opening and closing of door), and radio-frequency identification (RFID) tags that identify objects and people to track their locations [79].

As another hardware component, actuators are required to respond to commands or feedback from residents or to perform curated service actions decided from computing intelligence (to be explicated in the Datascape section), such as the control of ambience or home appliances [2].

**Table 1:** Smart servicescape elements in smart homes for health care services.

Smart servicescape categories				Smart servicescape elements in SHHSs^a^	Literature source
**Perceptible servicescape**					
	**Physical scape**				
		**Hardware components**			
			Sensors	Physiological sensors; environmental sensors; multimedia sensors; binary sensors	[2,3,12]
			Actuators	Actuators for device control	[2]
		**Environmental cues**			
			Ambience cues	Light; sound or noise; scent or odor; temperature and humidity; ventilation and radiation	[2,12]
			Object-state cues	Equipment status (eg, home appliances); space status (eg, home security and energy management)	[16]
		**Human states**			
			Physiological cues	Body temperature; weight; blood pressure and pulse rate; blood glucose; respiration and sweating	[12,16]
			Location or movement cues	Resident’s location and movement; other person’s location	[3]
			Activity or behavior cues	Habitual or regular activities; abnormal behaviors	[3]
	**Social scape**				
		Service relationship		Professional caregivers; clinicians at health care centers^b^; doctors and nurses at hospitals; staff at security or emergency centers^b^; staff at local welfare or community service centers^b^	[3]
		Social relationship		Informal caregivers (eg, family members and neighbors)	[3]
**Imperceptible servicescape**					
	**Datascape**				
		**Computing intelligence**			
			Data collecting	Resident health or activity monitoring; home environment (safety and security) monitoring; data patternizing^b^	[12,22]
			Data reasoning	Activity recognition and behavior prediction; anomaly and emergency detection	[2,3,22]
			Service curating	Home environment assistance; alert and emergency management; telemedicine service	[12,22]
		**Database**			
			Primary database	Environmental data; physiological data	[2,3,12]
			Secondary database	Situation or context data^b^; activity data and behavioral patterns	[3]
			External database	Clinical database^b^; cloud service^b^	N/A^c^
		**Communication network**			
			In-home network	Body area network; personal area network; wireless sensor network; local area network	[2]
			Bridging platform	Internet; mobile communication	[2]
			Secured communication channel	Security-related technology	[2]

^a^SHHSs: smart homes for health care services.

^b^These elements were newly added. Most of the paper sources for the elements of smart servicescape were literature review papers. The terms of elements were attuned to maintain consistent terminology, and the category labels were newly established in this study.

^c^N/A: not available.

#### Environmental Cues

The second category of the physical scape, environmental cues, is composed of *ambience cues* and *object-state cues*. Ambience cues include light, sound/noise, scent/odor, and air quality (ie, temperature, humidity, ventilation, or radiation), and they are usually detected by environmental sensors. Object-state cues involve (1) the equipment status (eg, the opening/closure or on/off of home appliances and the location of personal items) [3,12] and (2) space status (eg, home security or energy consumption status) [22], which are normally detected by binary sensors. Ambience cues and object-state cues captured by pertinent sensors are collected as input data for home environment monitoring, and they can also appear as output results of *service curation by computing intelligence* (to be explained in the section of Datascape). For instance, an increase in a bathroom’s humidity level, the opening and subsequent closure of the bathroom door, and an increase in water consumption can all be interpreted as the resident’s shower (or bath)-taking activity. Consequently, monitoring service functions can be activated to prepare for possible emergencies, such as slips or falls. Although the data collected from environmental cues may be straightforward, the interpretation to capture the full activities or behavior patterns requires additional knowledge of the environment and context.

#### Human States

Human states are essential elements as a target of health care services with real-time long-term health monitoring. Physiological cues, location/movement cues, and activity/behavior cues constitute this category. First, physiological cues are captured by diverse physiological sensors and collected as biometric data. Body temperature, heart rate, blood pressure, respiration rate, and body weight are the frequently monitored basic parameters [72,73]. Blood glucose can be regularly monitored for diabetic patients; oxygen saturation of blood or sweating can be measured, for instance, to monitor an elderly resident’s physical status during remedial exercises. Second, location or movement cues can also be detected by multimedia sensors (eg, video cameras) or binary sensors (eg, presence or motion sensors and pressure sensors). For example, when the resident is in a bedroom, his or her location can be detected by a closed-circuit television camera or a motion sensor installed in the room’s ceiling or more simply by pressure sensors equipped in a bed or foot mat. Location cues can be tracked for the individual that is being cared for or multiple residents, including other family members or cohabitants. The location of a third person (eg, visitors) can also be considered depending on service functions. Finally, activity/behavior cues can be considered advanced data derived from the interpretation of the former physiological cues and/or location or movement cues combined with environmental cues. For instance, a cooking activity can be detected combining the resident’s location in the kitchen by pressure sensors on a kitchen mat, his or her movement by a motion sensor in the kitchen, the opening/closing of a refrigerator door, the on/off of a gas stove switch, and so on [3]. In this way, habitual/regular activities, such as cooking, dining, sleeping, resting, or bathing, can be detected. At the same time, abnormal behaviors, such as slips or falls, can be captured based on irregular events, such as a sudden impact on a foot mat or an unusual duration of standing in locations [6,26].

### Social Scape

#### Service Relationship

In the circumstances of SHHSs, the residents themselves are the very target of health monitoring service. Therefore, they are included in the Physical scape as the source of human states. Meanwhile, the resident can interact with diverse stakeholders of health care services in service relationships. Professional caregivers visit the resident’s home regularly or irregularly to provide professional care services. Clinicians at health care centers are those who directly monitor the residents’ health status and provide consultations to maintain the resident’s health. Doctors and nurses can also be contacted through a telemedicine system; they may be interested in receiving updates about the progress of the resident’s disease based on health-monitoring data [3]. Meanwhile, other service relationships exist that are not directly relevant to health or medical services. When emergencies or security problems occur, the staff at security or emergency centers can be contacted by the residents or family members, or they can be automatically informed by an intelligent SHHS system detecting abnormal behavior. Staff at local welfare centers or community service centers may also be a part of nonmedical service relationships, and their visits to a resident’s home are important to check the living conditions of elderly people who live alone.

#### Social Relationship

In addition to service relationships, social relationships can be included in the social scape for those who work as *informal caregivers*. For instance, family members who live apart from the elderly resident, friends, neighbors, or any acquaintances can build informal relationships by routinely or irregularly contacting the elderly residents or by visiting them. Such relationships could be meaningful because the informal caregivers can help elderly residents engage in affective relations with emotional support for their daily lives.

### Datascape

#### Computing Intelligence

As previously noted, the Datascape is a distinctive characteristic of smart services as an imperceptible servicescape, and *computing intelligence* is a foremost part of Datascape that can make health care services *smart* by intelligent processing. Computing intelligence is composed of 3 elements, data collecting, data reasoning, and service curating, and the computing process in SHHSs also follows this order. Meanwhile, it seems that those elements of computing intelligence might be considered more as functions rather than servicescape elements. However, Datascape is considered an imperceptible environment in the SSW, so the collecting and processing of data that are inevitable parts to constitute the Datascape will also be regarded as environmental elements in this conceptual framework.

First, *data collecting* is an initial phase in which primary data related to human states (ie, physiological cues and location/movement cues) or environmental cues (ie, ambience cues and object-state cues) are gathered. The collected data are patternized and stored in a database to be analyzed for the next reasoning phase. For instance, data from sensors that detect environmental cues can be patternized using the following format: *Detection [Date] [Hour] [Sensor number] [Object code] [Location code]* [3]. Subsequently, the collected primary data are used to (1) monitor the residents’ health status and activity and (2) monitor the safety and security of the home environment.

Second, *data reasoning* uses a variety of knowledge engineering and data processing algorithms [3,12], such as artificial neural networks [80], fuzzy logic [81], hidden Markov models [82], and context-based reasoning [83] to analyze the primary data. The aforementioned algorithms are used to learn and develop models for the resident’s behavioral and physiological patterns as well as the home environmental patterns [2]. Consequently, data reasoning aims to (1) recognize the resident’s daily activities, (2) predict the resident’s behaviors based on activity recognition, and (3) detect anomalies or emergencies as the activities are carried out [3,22,84,85].

Finally, *service curating* is a substantial element of computing intelligence because it implies the final service solutions in SHHSs that have been generated from extensive interactions among the smart servicescape elements. Moreover, the service curating elements are directly experienced and perceived by the resident as *health care services*. According to the process of data reasoning and the decision-making process [2,26], computer intelligence can curate final solution services, such as (1) home environment assistance (eg, temperature control, gas/power control, or door opening/locking), (2) alert/emergency management (eg, a reminder to take pills, a warning when the gas is on, or an emergency call to family members/care centers), and (3) telemedicine service (eg, a progress check of chronic diseases, a consultation of health training status, or a prescription for medications).

#### Database

As the second category of the Datascape, the database is composed of a primary database, secondary database, or external database. First, the primary database stores environmental data and physiological data directly collected from relevant sensors or devices. It is usually a local database, and it provides those data to central servers—the computing intelligence—as inputs for further processing. Second, the secondary database accumulates the analyzed output data from the computing intelligence, such as situation/context data, activity data, and behavioral patterns. As the secondary database becomes strengthened by data accumulation cycles, the recognition accuracy of activities or behavioral patterns can be augmented by machine learning through computing intelligence [3,86,87]. Finally, the external database can be accessed by the database of SHHSs with authorized personnel’s permission, for instance, to access a clinical database such as personal electronic medical records (EMRs) from health care centers. Moreover, a generic database such as cloud services can be accessed and utilized by SHHSs.

#### Communication Network

The sensors and actuators of the hardware components in SHHSs are connected with computing intelligence and the database through a communication network [3,12]. Consequently, the environmental cues and the human state cues captured by the sensors are transmitted to the central computing server over a wired and/or wireless communication medium [2].

*In-home network* refers to the communication platform that provides data communication inside the smart home. Body area network (BAN), personal area network (PAN), wireless sensor network (WSN), and local area network (LAN) can be included in this category, in order of range [2,12]. Physiological sensors integrated in wearable devices usually worn by the resident form the BAN. In addition to the wearable on-body sensors, diverse sensors on personal devices can be connected through RFID or Bluetooth, forming the PAN. The BAN or PAN can be connected with other environmental sensors and actuators through the WSN (eg, ZigBee) [88]. The central platform of computing intelligence can communicate with any sensors and actuators in the smart home using the WSN to collect data or send feedback for pertinent actions [2]. Consequently, those short-range and low-powered communication platforms of in-home networks form the LAN using a technology such as Wi-Fi.

Moreover, bridging platforms, such as the internet and mobile communication, are cost-effective and readily available solutions for remote communication to access the external database [12] and transmit various types of data, such as text, image, voice, and video [3]. Furthermore, secured communication channels [12] are required particularly for health care services to transmit data to/from the clinical database, such as personal EMRs in health care centers, because of privacy and authentication issues [89].

## Discussion

### How to Apply the Smart Servicescape Wheel

#### Implications for Service Planning and Development

As previously noted, the value of adopting the ecological approach by proposing the SSW lies in delineating the extensive spectrum of environmental constraints in the complex adaptive system of SHHSs. Diversified and undefined service experiences can be generated by multifaceted combinations of smart servicescape elements that could influence and shape the domain of users’ behavior. Therefore, the SSW defined the smart servicescape elements at the level of components or functions. Founded on this conceptual framework, it would be worthwhile to suggest how to apply the SSW in research or practice for the development of SHHSs.

First, the SSW can be utilized to establish requirements for developing SHHSs. When service planners and developers contemplate elements to combine to provide appropriate service functions and service contexts, the SSW can serve as a *map* or a *list of candidates* to demonstrate various and possible options for smart servicescapes. It can help service planners and developers to consider the various types of perceptible servicescape elements—namely, the types of sensors or actuators and the types of information (eg, information from environmental cues, human states, or social scape relationships)—and their diverse combinations. Associated with their types and combinations, the types of imperceptible Datascape elements would be determined, such as the types of input or output data in terms of database, the types of data modeling and decision-making algorithms regarding computing intelligence, and the types of service curation. Moreover, depending on the service curation types, the pertinent elements of a Physical scape or Social scape can be selected for activation in response to planned actions.

Second, the SSW can be applied in association with work domain analysis (WDA). Founded on the ecological approach, WDA is a part of cognitive work analysis that identifies the functional structure of a system (ie, a work domain) independent of activities [63,90,91]. WDA’s worth lies in the fact that the separation of structure from activities helps bring an important source of order to the analysis of complex adaptive systems such as SHHSs [92]. The WDA requires the determination of physical resources, technical functions, domain functions, domain values, and system purpose [92]. Therefore, the SSW can provide candidate elements for the layer of physical resources—and partially includes the layer of technical or domain functions in case of the elements of computing intelligence. In accordance with the specific properties of service concepts in SHHSs, service planners/developers can select relevant smart servicescape elements and connect them to pertinent functions and values to realize an intended service purpose effectively. Conversely, based on the various combinations of smart servicescape elements, innovative functions or values of service domains can be newly defined, and a novel service system can be proposed.

Finally, the SSW can contribute to the understanding of real contexts in SHHSs and the anticipation of diversified service experiences. The extensive spectrum of environmental elements illustrated by the SSW can support the anticipation of possible behavioral patterns in the resident’s service experiences. Diverse combinations of the smart servicescape elements and their subsequent patterns of user behaviors can be accumulated in the database and learned by the computing intelligence employing machine learning technologies. Consequently, this process can improve the accuracy of behavior prediction and enhance the appropriateness of service curation in SHHSs.

#### Implications for Service Management

From the perspective of service management, the SSW can be utilized to develop measurement items for the operation and evaluation of SHHSs. To determine the items to evaluate services, the SSW can serve as a comprehensive list of elements to be considered. If the detailed evaluation measurement scales are developed, it could be applied in various ways such as to (1) diagnose the current state of service operation, (2) determine improvement points by identifying weaknesses in terms of components, functions, and services, (3) enhance the pertinence of actions against unexpected events or service failures, and (4) strengthen the thoroughness of SHHS management. Moreover, this kind of evaluation can find an opportunity to extend its application domains toward other services based on SHHSs.

### Limitations and Future Research

Despite the value and opportunities presented by the SSW, several limitations of the framework stem from its focus and conceptual nature. As noted in the Introduction, service experiences could be cocreated by multidimensional association among 3 layers: servicescape, service interactions, and service contexts. However, the focus of the SSW is limited in describing detailed servicescape elements rather than in determining the interactions that occur between those elements or defining probable influences from/on social and cultural structures (ie, service context). For the sake of service planning and development, the other 2 layers of service interactions and service contexts need to be profoundly investigated.

First, in terms of the service interaction layer, the next phase after defining the environmental components and functions could be the establishment of detailed interactions and activities shaped by the combination of smart servicescape elements. Direct interactions such as those between the service user (ie, the elderly resident) and the perceptible servicescape need to be identified. Furthermore, the *ambient interactions* among the imperceptible and perceptible servicescapes (ie, the Datascape, Physical scape, and Social scape) without the user’s direct intervention require a more comprehensive exploration considering the ambient characteristic of smart services. The layer of service interactions might allow us to consider innovative ways of direct and ambient interactions for more natural and user-centered experiences of SHHSs.

Second, regarding the service context layer, social and cultural structures such as social norms and institutions could influence the ways of service interaction and experience formation. Particularly, social structures and institutions pertinent to SHHSs need to be investigated in terms of ethical and legal issues because personal data collected through smart home technologies require a great degree of protection [17,20]. Therefore, norms, regulations, policy, and legislation systems in association with privacy, security, and confidentiality issues in SHHSs would require high attention in future research [93].

Third, as the SSW is a theoretically established conceptual framework, further empirical studies to evaluate the validity of the framework are needed; it should be applied in the development of a new system of SHHSs or in the analysis of real use cases of SHHSs. Moreover, because the SSW is defined at the conceptual component or function level, it is limited in its elucidation of real products or systems that could be the complete combination of smart servicescape elements. Therefore, further research on the association of the SSW components to describe the cases of actual products or systems could be possible.

Finally, although the aspect of Social scape was covered less extensively than other scape categories in this study, it does not mean that it is less significant. The Social scape has been investigated largely from the perspective of stakeholders in service research, and the stakeholders in SHHSs would be complicated considering their intricate relationships. Therefore, it could be required to explore the interplays of Social scape parties in association with the elements of Physical scape and Datascape.

### Conclusions

This study asserted the value of an ecological approach with emphasis on environmental constraints for understanding SHHSs. This study is based on 2 rationales: (1) users tend to perceive service quality and service experiences through the servicescape because of the intangibility of health care and the pervasiveness of smart home services and (2) both service domains are complex adaptive systems in which diversified and undefined service experiences—not only a few intended service flows—can be generated by complex combinations of servicescape elements.

Accordingly, the conceptual framework of the SSW was proposed as an ecological approach delineating the extensive spectrum of environmental constraints in SHHSs. Generally divided into perceptible and imperceptible servicescapes, the SSW consists of the perceptible Physical scape (ie, hardware components, environmental cues, and human states), the Social scape (ie, service relationships and social relationships), and the imperceptible Datascape (ie, computing intelligence, database, and communication networks).

The strengths of the SSW lie in its various application opportunities. The SSW can be utilized in service planning and development to (1) establish the requirements for SHHS development, (2) associate with WDA by defining component layers, and (3) understand the real contexts of SHHSs to enhance the prediction of diverse service experiences, as well as in service management to develop measurement items for the operation and evaluation of SHHSs.

## Abbreviations

BAN: body area network
EMR: electronic medical record
HCI: human-computer interaction
IoT: internet of things
LAN: local area network
PAN: personal area network
RFID: radio-frequency identification
SHHS: smart homes for health care service
SSW: Smart Servicescape Wheel
WDA: work domain analysis
WSN: wireless sensor network
